# Silver and Gold Nanoparticles for Antimicrobial Purposes against Multi-Drug Resistance Bacteria

**DOI:** 10.3390/ma15051799

**Published:** 2022-02-27

**Authors:** Navid Rabiee, Sepideh Ahmadi, Omid Akhavan, Rafael Luque

**Affiliations:** 1 Department of Physics, Sharif University of Technology, Tehran 11155-9161, Iran; oakhavan@sharif.edu; 2 School of Engineering, Macquarie University, Sydney, NSW 2109, Australia; 3 Student Research Committee, Department of Medical Biotechnology, School of Advanced Technologies in Medicine, Shahid Beheshti University of Medical Sciences, Tehran 19857-17443, Iran; speahmadi@yahoo.com; 4 Cellular and Molecular Biology Research Center, Shahid Beheshti University of Medical Sciences, Tehran 19857-17443, Iran; 5 Departamento de Química Orgánica, Campus de Rabanales, Universidad de Córdoba, Edificio Marie Curie (C-3), Ctra Nnal IV-A, Km 396, E14014 Cordoba, Spain

**Keywords:** silver nanoparticles, gold nanoparticles, antimicrobial resistance, green chemistry

## Abstract

Several pieces of research have been done on transition metal nanoparticles and their nanocomplexes as research on their physical and chemical properties and their relationship to biological features are of great importance. Among all their biological properties, the antibacterial and antimicrobial are especially important due to their high use for human needs. In this article, we will discuss the different synthesis and modification methods of silver (Ag) and gold (Au) nanoparticles and their physicochemical properties. We will also review some state-of-art studies and find the best relationship between the nanoparticles’ physicochemical properties and potential antimicrobial activity. The possible antimicrobial mechanism of these types of nanoparticles will be discussed in-depth as well.

## 1. Introduction

Bacterial resistance is becoming a global challenge as microbes are continually changing. Every year, 33,000 people in Europe die from bacterial resistance, so new ways to fight bacteria are required [1]. The increase in nosocomial and acquired infections can be a significant problem due to multi-drug-resistant bacterial pathogens (MDRs) for which current antibiotic treatments are not effective [2]. Antibiotics are important in fighting bacterial infections, however, in recent years they have become increasingly resistant to treating infections. Excessive application of antibiotics causes microorganisms to undergo genetic changes over time to live antimicrobial use leading to antimicrobial resistance (AMR) [3]. Excessive usage of antibiotics and the progress of antibiotic resistance has become a global concern. The increase in nosocomial infections and viral infections, including influenza as well as SARS-CoV-2 in 2019, requires rapid risk assessment and immediate prevention and treatment. Recent research has warned of a link between SARS-CoV-2 and AMR [4,5].

AMR describes the resistance of any microbe to the drugs that are applied to kill them. Drug-resistant diseases cause about 0.7 million deaths a year, and if no attempt is made to control it, AMR deaths, at worst, could reach 10 million a year by 2050 [6]. As a result, it can be said that AMR is one of the most important threats to human health and causes an increase in mortality. AMR is a multipart apparatus whose etiology can be influenced by the individual, the bacterial strains, and the resistance mechanisms that are established.

The mechanism of AMR includes the restrictive uptake of a drug, inactivating a drug, altering a drug target, and active drug efflux [7]. Additionally, AMR has been reported at three increasing levels including extensive drug resistance (XDR), MDR, and pan-drug resistance (PDR) [8]. XDR was determined as non-susceptibility to, at minimum, one agent whenever two or fewer antimicrobial types were listed. PDR was determined as non-susceptibility to all agents in all antimicrobial types. MDR was determined as attained non-susceptibility to at least one agent in three or more antimicrobial types [9,10]. To overcome the problem of resistance, it will be essential to alter the protocols of applying antimicrobials so that these drugs are administered when all other treatment routes have failed.

Nanoparticles (NPs) can offer an alternative possible solution to battle MDR pathogens [11,12]. In fact, NPs with a size of less than 100 nm show a higher toxicity due to their exclusive possessions [13,14]. Several NPs, including silver (Ag) and gold (Au) NPs (and also copper (Cu) NPs [15,16], zinc (*Zn*) NPs [17,18], Nickel (*Ni*) NPs [19], *platinum* (Pt) NPs [20], and palladium (Pd) NPs [17,19]), have exposed different antimicrobial activities based on the physicochemical properties of each metal nanomaterial. Among these, Ag NPs showed more antimicrobial activities using multiple mechanisms. In addition to the synthesis method, the size and shape of Ag NPs show an important role in this activity. Interestingly, several reports show the high antimicrobial activity of Ag NPs with smaller sizes. These NPs can disrupt the bacterial cell membrane, affecting cell penetration and causing toxicity [21]. The antibiofilm activity of Ag NPs has been also shown in several studies. According to the properties of Ag NPs, they can improve the antibiofilm activity of conservative antimicrobials [22,23].

Au NPs are one of the significant branches of nanometal research, especially in antimicrobial activity [24]. Determinants of the antimicrobial effects of Au NPs are broadly studied, such as shape, size, concentration, and coating agent. Due to the electrical and optical properties of Au NPs, they have more consideration. One of the predominant properties is their localized surface plasmon resonance (LSPR), which shows a significant role in various applications such as biosensors. This occurs as the electrons on the surface of noble metal NPs interrelate with electromagnetic radiation, producing LSPR; it is because of this that metal NPs create strong extinction, useful in various scenarios [25]. Au NPs have been known to have unique properties to LSPR biosensors due to their spectral response to the location of the NP’s surface [26].

The antibacterial activity of Au NPs can also be classified through the photothermal properties of Au NPs or their uses in photodynamic therapy (PDT) technology. Phothermal therapy (PTT) refers to transforming light into thermal energy by gold NPs. Gold nanorods (GNRs) and nanostars (GNSs) are used for this assay to eliminate bacteria. The PDT method is based on irradiating photosensitizers which produce reactive oxygen species (ROS) and therefore destroy bacteria [25,27]. Conjugating Au NPs with antibiotics, antimicrobial peptides (AMP), and other ligands have a tendency to enhance the antibacterial abilities, decrease the requirements of high doses, and diminish side effects [25]. Au NPs and Ag NPs were applied as drug delivery systems in antibiotics and AMPs [28]. Surface modification of the ligands can affect the antimicrobial efficiency of Au NPs [29]. Functionalized Au NPs with antibiotics or drugs show increased antimicrobial activity than antibiotics alone. In fact, Au NPs conjugated with biomolecules reduce bacterial cell growth [30]. Functionalized Au and Ag NPs with smaller sizes have various benefits in drug and antibiotic delivery such as regulating size and morphology, high-density surface ligands, and delivery without losing drugs which protects them from destruction [31].

The purpose of this article is to investigate the role of Au and Ag NPs in antimicrobial resistance and their effects. These nanomaterials are crucial in antimicrobial applications for several reasons: first, they have a large surface area, optical properties, and are made of cost-effective materials, enabling great synergy in antimicrobial actions. Second, they can target the structure of bacteria, and indications have revealed that the synergistic act of NPs and antibiotics triggered an increase in the antibacterial potential of antibiotics. We provide an overview of the unique properties of Au and Ag NPs used in the destruction and inactivation of AMR and MDR bacteria, the advance of biosensors, and their use as drug delivery systems, as well as addressing the challenges related to the use of these noble metals in this field and their possible solutions.

## 2. Methods for the Synthesis of Au and Ag NPs

**Figure 1:**
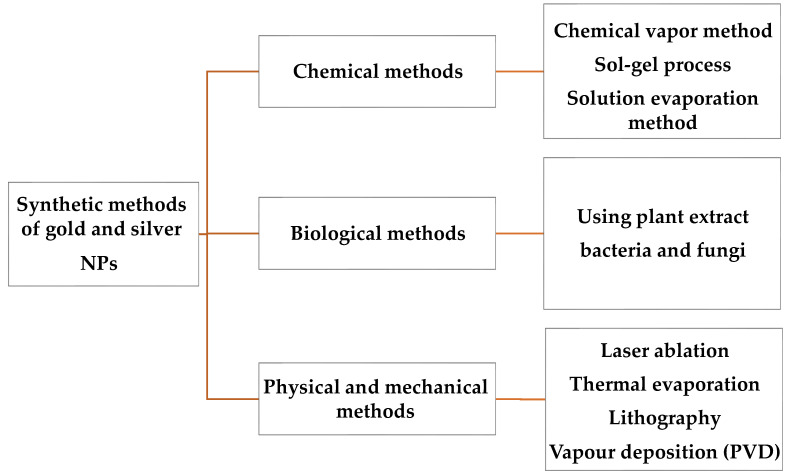
Various synthesis methods of Ag and Au NPs.

Various methods such as biological, physical and mechanical, and chemical and photochemical can be applied to synthesize Ag and Au NPs [32] (Figure 1). A chemical assay reduces Ag^+^ to Ag^0^ through a reducing agent, including sodium borohydride in the existence of a stabilizer, to preserve Ag NPs from aggregating. These reducing agents could be designated cautiously to restrict environmental influence while increasing particle stability [33].

### 2.1. Chemical Methods

There are various methods for making Au NPs, the simplest of which is to reduce gold salt in the existence of a reducing agent [34]. The formation of Au NPs begins in the nucleus of Au ions. To avoid aggregation, a stabilizing agent is usually added through the synthesis procedure. This assay was first introduced by Turkevich in 1951 [35]. Au NPs are formed by the reduction of gold salts in water using citrate ions as a reducing agent which results in the production of spherical NPs with a diameter of 20 nm [36]. Sodium citrate in this process acts as both a reducing agent and a stabilizing agent through adsorption on the surface. Frances then stated in 1973 that Au NPs can be obtained in different sizes by controlling the reducing agent/stabilizing agent ratio [37]. Accordingly, Au NPs have recently been developed with the simultaneous addition of citrate salts and a surfactant such as sodium-3 mercaptopropionate [38]. Because the reactivity of NPs is very high and they tend to precipitate, a stabilizer (coating) is added to them to preserve their desirable properties [39]. Co-sputtering of Au and SiO_2_ followed by post-annealing has been reported as one of the physical methods for the fabrication of Au NPs [40]. The Brust-Schiffrin assay was developed through Brust and Schiffrin in 1994. This proved to be an easy method for the synthesis of thermally stable Au NPs of controlled size [41].

Synthesis of Ag NPs can be performed by various chemical methods, including sol-gel, which is one of the most common chemical methods [42]. However, different advantages and limitations were observed related to these techniques. For example, they need a high amount of energy and a tube furnace at atmospheric pressure [32]. Synthesis of Ag NPs with a size of ~10 nm can be performed by thermal decomposition [43], low-temperature self-agglomeration of sol-gel films [44], and sputtering/post-annealing the Ag layer deposited on a TiN buffer layer [45].

### 2.2. Photochemical Processes

Photochemical procedures can be used in the synthesis of metallic NPs, including AuNPs and Ag NPs, according to the improved temporal control these methods propose. The process involves exposing solutions comprising the metal precursors to visible or ultraviolet (UV) light. This method is beneficial as it evades the application of toxic compounds, does not need high-cost equipment, and can be performed at RT conditions as opposed to other methods, such as chemical approaches [46,47]. The photochemical procedure initiates with the reduction of the precursor of metal from n^+^ valence (M^n+^) to its zero-valence state (M^0^) through the photocatalyzed act of the reducing agent. Then, the M^0^ creates nucleation cores that grow and aggregate to provide the metallic NPs [48]. In this method, the type of light source, such as UV or visible light, and the irradiation time and application of stabilizing agents are important factors that impact the properties of the acquired NPs [49].

UV radiation with wavelength ranging from 100–400 nm can be used in this method. The UV-mediated photochemical synthesis of Ag and Au NPs is an effective method. It shows various properties, such as the use of water as a solvent, and the use of non-toxic capping agents that confirm NP stability. These experiments can be performed at RT conditions. In general, silver nitrate (AgNO_3_) is applied as the precursor and polymers are applied as stabilizers [50,51].

### 2.3. Physical Methods

Synthesis of Ag NPs can be performed by various physicochemical methods, including laser ablation and evaporation-condensation, which are the most significant physical approaches. The uniformity of NP distribution and lack of solvent contamination in the synthesized thin films are the benefits of the physical synthesis methods over chemical methods [32].

Ag NPs could be prepared through laser ablation of metallic bulk materials. In fact, the efficacy of synthesized Ag particles is determined by various factors such as the wavelength of the laser impinging the metallic target, the liquid medium, and the period of the laser pulses [32,52].

Laser ablation can also be applied to synthesize Au and Ag NPs; the properties of the NPs produced rely on the laser wavelength, duration of the laser pulses, and liquid environment [53,54]. Microwave irradiation is one physical technique for the synthesis of Au NPs through reducing agents, including citric acid, and a binding agent, including cetyltrimethylammonium bromide (CTAB) [55,56]. The γ-irradiation assay is also a method for the synthesis of Au NPs with identical size of about 10 to 40 nm with high purity using polysaccharide alginate as a stabilizing agent [57,58,59]. However, techniques based on physical vapor deposition, including laser ablation, need a comparatively expensive apparatus, however, the variability of the synthesis is large, and a broader spectrum of NPs may be prepared, particularly in the case of PVD techniques where fewer chemicals are needed [60].

### 2.4. Biological Methods

The synthesis of Au and Ag NPs by green assays is more improved than chemically synthesized Ag NPs concerning antimicrobial activity. In the green synthesis method, nanoparticles in the size range of 1 to 100 nm are formed by bottom-up technology [61]. In this method, various types of fungi, bacteria, plants, yeasts, etc. are applied [62,63]. Recent studies show that Ag NPs synthesis using bacteria is inexpensive, easy, and attractive; these factors have managed to improve the number of outcomes of Ag NPs synthesis with various gram-positive and gram-negative species. The Ag NPs produced by this green method have a smaller average size, more stability, better size distribution, and higher production efficiency than the particles produced by modified microbial and polysaccharide methods. Additionally, the production time of NPs in this method is lower than the microbial method and the production cost is lower than the modified polysaccharide method [64,65].

High cost and environmentally friendly assays have recently appeared mostly by biological outcomes to the synthesis of nanostructures with exclusive antimicrobial properties. To this end, the biosynthesis of Au and Ag NPs was done using the cell-free extract of red yeast *Phaffia rhodozyma* [66]. Conversion of metal ions to NPs in a friendly one-step method can be done by biological molecules in plant and yeast extracts.

## 3. Ag Nanoparticles for Antimicrobial Resistance

The antimicrobial effects of Ag NPs have been studied and their operative potential against a different range of microbes, including antibiotic-resistant bacteria, has been demonstrated. Dead bacteria have been observed by imaging and elemental analysis using transmission electron microscopes (TEM), scanning electron microscopy, and EDX (X-Ray Probe Micro Analyzer). Additionally, researchers have concluded that Ag NPs react with elements of bacterial cell membrane structures and lead to cell damage. Ag NPs also have antibacterial activity against gram-positive and gram-negative bacteria, but according to conflicting reports, gram-negative bacteria are more sensitive to Ag NPs than gram-positive bacteria [67,68,69]. Ag NPs show antimicrobial activity by various mechanisms. Ag NPs two main killing pathways are known: interaction with the microbial membrane and disruption of the functioning of the membrane. Interaction of the positively charged Ag NPs with the negatively charged bacterial cell wall can cause changes in the cell wall, resulting in increased cell permeability leading to cell death. The tendency of Ag NPs to react with phosphorus-containing sulfur molecules in extracellular membrane (membrane proteins), and intracellular components (DNA and proteins) is high, which can have an important effect on cell proliferation. Moreover, the creation of reactive oxygen species (ROS) near bacteria and inside of them increases reasons for oxidative stress. The interaction of Ag NPs with the thiol group can lead to the induction of ROS, inhibition of respiratory enzymes, and apoptosis of cells [70,71].

Research has also investigated the role of Ag NPs and the mechanisms of antimicrobial activity exerted by Ag NPs (N-Ag). Efflux pumps can export the antimicrobials agents out of the cell before they can spread their target sites. Resistance to an antimicrobial agent can be acquired or inherent. Bacteria can also obtain new resistant genes from other bacteria and integrate them into their chromosomes. Therefore, due to the proliferation of multidrug-resistant microbial strains, the use of Ag NPs as an antimicrobial to control antimicrobial strains should be considered [72,73].

Different research has described the antimicrobial activity of Ag NPs against AMR microbes. The antibacterial activity of Ag NPs with a concentration range of 30–100 mmol/L were effective against erythromycin-resistant *E. coli*, *Streptococcus pyogenes*, and *Pseudomonas aeruginosa* (*P. aeruginosa*) [74].

Researchers assessed the antibacterial activity of Ag NPs on *P. aeruginosa* by classification of the proteomic response. Ag NPs can be activated by interaction with the membrane and the production of ROS. In fact, the higher the ROS production in the cell, the higher the oxidative damage caused by Ag NPs, so the antimicrobial activity of Ag NPs can be adjusted according to the increased release of Ag ions in solution [67].

Ag NPs can exhibit potent antimicrobial activity with MIC in the range of 1.4–5.6 µg/mL and MBC in the range of 2.8–5.6 µg/mL. These nanoparticles disrupt the structure of bacteria and increase the level of alkyl hydroxide reductase which increases the production of ROS and increases apoptosis [75].

Another study exhibited the green synthesis of Ag NPs through the aqueous leaf extract of the seasonal desert plant *Sisymbrium irio* against different MDR bacteria. TEM images exhibited a particle size of 24–50 nm. According to the results, different concentrations of Ag NPs were tested against MDR *P.aeruginosa* and *Acinetobacter baumanii* and *Escherichia coli* (*E. coli*) used as controls. Ag NPs exhibited real inhibition of *P. aeruginosa* and *Acinetobacter baumanii* at the concentration of 6.25 µg, and the concentration of 100 μg showed effective antibacterial activity on *P. aeruginosa, E. coli,* and *Acinetobacter baumannii* (*A. baumannii*). As a result, Ag NPs could act as operative agents to battle MDR microbes [76].

The green synthesized Ag NPs using endophytic *E. hormaechei* show major antimicrobial activity against *B. cereus*, *C. albicans*, *S. aureus*, and *S. aureus*. Spherical and polydispersed Ag NPs have a size of about 9–92 nm within 5 min, and these NPs were stable with a mean ζ value of about −19.73 mV. In this assay, Ag NPs synthesis was quick and eco-friendly; compared to traditional antibiotics, the Ag NPs were highly operative against *B. cereus*, showing about 9 mm and 8 mm zones of inhibition (ZOIs), respectively. These results suggest that these Ag NPs are capable antimicrobial agents that can be applied to the process and formulation of novel drugs to reduce the AMR in pathogenic and MDR microbes [6].

Researchers used *Handroanthus impetiginosus* extract as a reducing and capping agent in the synthesis of Ag NPs by the microwave technique. Bactericidal activity of the NPs was confirmed against *E. coli* and *S. aureus* establishing high inhibition possible to both bacterial strains with a MIC of 6.7 × 10^4^ μg/mL and 3.1 × 10^2^ μg/mL, respectively, though the extract showed a low MIC in both bacteria. Remarkably, the bactericidal activity of Ag NPs is greater in *S. aureus* than *E. coli*. This was related to the mixture of Ag NPs with the capping layer comprising natural compounds with antimicrobial characteristics and showing high antibacterial effects compared to gram-negative strains [77].

Ag NPs can be applied in the inhibition and killing of planktonic bacteria, as well as successfully inhibiting biofilm formation [78]. Although, naked Ag NPs have a tendency to aggregate because of their great surface energy resulting from their large specific surface area they most certainly oxidize in storage [79]. However, different Ag NP-based composites have been synthesized including magnetic NPs (MNP)@Ag NPs, CNT-Ag NPs [80], graphene oxide(GO)-Ag NPs, and TiO_2_-Ag NPs [81,82] which can increase the permanency of Ag NPs, and offer antibacterial and antibiofilm effects other than naked Ag NPs [83]. In addition, it has been reported that by using electric field stimulation, the antibacterial activity of Ag nanostructures can be increased significantly [84].

The use of Ag NPs to prevent biofilm has been reported in several studies. Ag NPs synthesized using *Streptomyces* strains the size of 10–30 nm showed the ability to inhibit the growth of pathogenic strains such as methicillin-resistant tetracycline-resistant *Neisseria gonorrhoeae* and *Staphylococcus aureus* (*S*. *aureus*). It also reduced biofilm production by *Pseudomonas aeruginosa* by 80%. Another use of these nanoparticles can be using Ag NPs as a coating material on medical devices used to treat patients to prevent the creation of biofilms [85].

Spherical Ag NPs with a size of 30 nm showed high ability in preventing *P. aeruginosa* biofilm. Biofilm can be more resistant to Ag NPs compared to planktonic cells. If a high concentration of Ag NPs, about 18 μg/mL, were used, biofilm development was fully prevented. However, low doses of AgNPs can be late in preventing the growth cycle of biofilm. Additionally, sublethal doses of Ag NPs improved the manufacture of proteins and polysaccharides compared to a control, which meaningfully changed the biofilm structure [86].

High antimicrobial activity was shown by Ag NPs synthesized by *Terminalia catappa* leaf extract (TCE) via a one-pot single-step assay (TCE-Ag NPs) with different concentrations. Various concentrations of TCE have produced altered-sized Ag NPs. TCE-Ag NPs-3 show effective prevention of the biofilm formation of methicillin-resistant *S*. *aureus* (MRSA) and multi-drug-resistant *P. aeruginosa* (MDR-PA) by about 70% and 73%, respectively, at a concentration of about 8 µg/Ml. The results suggest that the treatment of 100 µL of TCE-Ag-1 can suppress the development of MRSA, MDR-PA, and *C. albicans* of about 11, 19, and 14 mm, respectively. Moreover, the diameters of growth inhibition zones with TCE-Ag-2 were initiated to be 12, 20, and 15 mm against MRSA, MDR-PA, and *C. albicans*, respectively. Additionally, the diameters of growth inhibition zones with TCE-Ag-3 were about 14-, 21-, and 17-mm in diameter against MRSA, MDR-PA, and *C. albicans*, respectively. These NPs can destroy the membranes of MDR-PA, MRSA, and *C. albicans* [87].

The combination of Ag NPs with organic molecules shows improved antimicrobial activity. For example, lignin, a hydrogel formulated with Ag NPs, was produced. The product exhibited great antimicrobial activity against *S. aureus* with about 100% of the bacteria killed after several hours of treatment. The platform showed high biocompatibility with L929 cells [87]. (Bio)-polymers can increase the antimicrobial activity of Ag NPs [88,89]. Chitosan was an appropriate polymeric material to encourage Ag NPs capping, which shows excessive microbial probability and decreases cytotoxic effects. Ascorbic acid chitosan and ascorbic acid were used as the capping agent and reducing agent, respectively, and were applied for the synthesis of Ag NPs with small size (<10 nm). The loading of Ag NPs and other agents onto the coating can release the molecules and kill pathogens. For example, the synthesis of chitosan –ascorbic acid–Ag nanocomposites was developed. It was established that the nanocomposites showed an important microbial activity against the *S. aureus*, *E. coli*, and *P.aeruginosa*, as well as the inhibition of biofilm development. These nanocomposites also showed a reduction in the induction of cytotoxicity in mammals. However, further doses of potential toxicity and drug resistance need to be investigated [88]. In general, controlled release can be a significant and efficient assay to prevent biofilm formation. Furthermore, in the formation of biofilm, the use of chitosan alone would not be suitable.

Using chitosan and brown algae extract, researchers synthesized Ag NPs that exhibited improved bactericidal activity against *Bacillus cereus* and *Salmonella enterica.* The antibacterial evaluations showed that Ag NPs synthesized with a combination of algae extract and chitosan showed greater bactericidal activity with ZOI values greater than 16 mm in all bacteria, although ZOI values of Ag NPs or extract alone were lower [90]. In general, Ag NPs with chitosan were incorporated throughout their synthesis procedure, and the formulations produced based on the NPs showed high antibacterial capacity.

An interesting point in this regard is the mixture of Ag NPs with antibiotics to improve antimicrobial activity. Katva et al. exhibited that the mixture of gentamicin and chloramphenicol with Ag NPs show a high antibacterial effect in MDR *E. faecalis* [91]. In another study, researchers exhibited that Ag NPs combined with other antibiotics can show a synergistic effect, displaying an improved antibacterial activity at concentrations less than the MIC of NPs or the antibiotic [92].

The use of Ag NPs is also involved in industry and commerce due to their antimicrobial properties. The use of Ag NPs in face masks can help improve their protective ability. A study by Li and a colleague showed the production of masks coated with Ag nitrate and titanium dioxide nanoparticles that were able to reduce up to 100% of *S. aureus* and *E. coli* in one day. As a result, the use of Ag NPs face masks can prevent infection in places, e.g., hospitals, where there is a high persistence of pathogenic microorganisms [93].

In general, Ag NPs show high antimicrobial activities. They address various conditions to which novel antimicrobial technologies are emerging and can be operative concerning antimicrobial activity and low cytotoxicity. The application of the Ag NPs reduces the doses of the antibiotic and the nanoparticle needed to attain an operative antibacterial activity against various MDRs microbes. These NPs can form complexes to perform as nanocarriers of antibiotics that improve their release and enhance their antibacterial outcome. Table 1 shows other examples of the application of Ag NPs in antimicrobial resistance activity (AMR).

## 4. Gold Nanoparticles for Antimicrobial Resistance

Unlike Ag, which has strong antimicrobial properties, Au is not recognized as having an inherent antimicrobial property. Although, the characteristics of nanoscale Au NPs can provide strong particle performance and scientists have discovered the possibility of using Au NPs in biofilm treatment. Au NPs can be synthesized by reducing gold salts through several completely fixed methods [105,106]. Moreover, Au NPs can also be synthesized by reducing gold salts through several completely fixed methods [105,106]. In recent years, Au NPs have been applied in various fields according to their optical properties and surface plasmon resonance [36].

The antimicrobial activity of Au NPs has developed a significant research area according to their exclusive physicochemical properties [107]. The use of chemicals and drugs can cause problems as therapeutic agents, including low specificity, insufficient cell penetration, and short half-life. Many of these problems can be solved in combination with Au NPs. For example, the improvement of the anti-angiogenic activity of MCF-7 cells by kaempferol- bounded Au NP compared to kaempferol alone [108].

**Figure 2:**
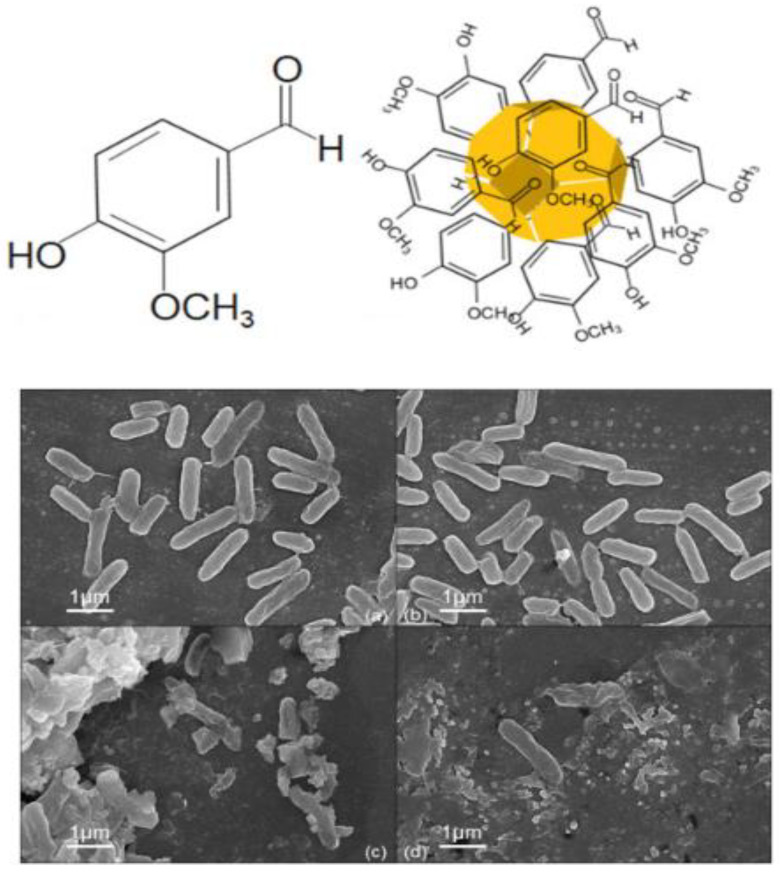
Schematic illustration of synthesized VAuNPs by vanillin (**the above illustration**); FESEM image of *P. aeruginosa* in untreated, in presence of the (**a**) 50 μg/mL of VAuNPs, (**b**) Meropenem-200 μg/mL, (**c**) Meropenem (20 μg/mL) and (**d**) VAuNPs (50 μg/mL) (**the below images**) [24]. Reprinted with permission from Elsevier.

The functionalized Au NPs with small molecules can provide improved bioavailability, stability, and biocompatibility than the carriers alone. Several irregular-shaped Au NPs can simply adsorb molecules on their surface, display a worthy plasmon resonance, and be used in the recognition of cancer cells [109]. The capping of Au NPs with vanillin (VAuNPs), which is synthesized by green assay, is used as an inhibitor of MexAB-OprM efflux pump components (Figure 2). Although VAuNPs show non-bactericidal effects at high concentrations, the antibiotic potential was considered in combination with seven applied antibiotics in contradiction of *P. aeruginosa*. The results showed that if about 50 μg/mL of VAuNPs were used, a decrease in the inhibitory concentrations of the antibiotics trimethoprim and meropenem was detected by 10 and 14 times, respectively. Au NPs in a mixture with trimethoprim and meropenem can also provide effects about 3-times better than naked vanillin. The results also show that the activity of the output pumps is blocked by VAuNP. Therefore, the large decrease in MIC of antibiotics was attributed to the suppressive activity of the VAuNPs outlet pump. It was stated that VAuNP increases sensitivity to the antibiotics meropenem, trimethoprim, and others. In general, the capability of VAuNPs and vanillin to be applied as antibiotic adjuvants to inhibit bacterial outflow pumps is used to boost antibiotics and address the AMR that affects human health and the environment [24].

Antimicrobial peptides (AMPs) are debated as elegant substitutes to traditional antibiotics in the battle against multi-drug resistance pathogens which can kill pathogenic bacteria, fungi, and protozoa, according to the broad-spectrum of activity [110,111,112]. Gu and colleagues, first reported the binding of AMPs bound to metal nanoparticles, such as Au, and exhibited that vancomycin bound to Au NPs (Au@Van) through thiol bonds can be active against vancomycin-resistant enterococci [113].

**Figure 3:**
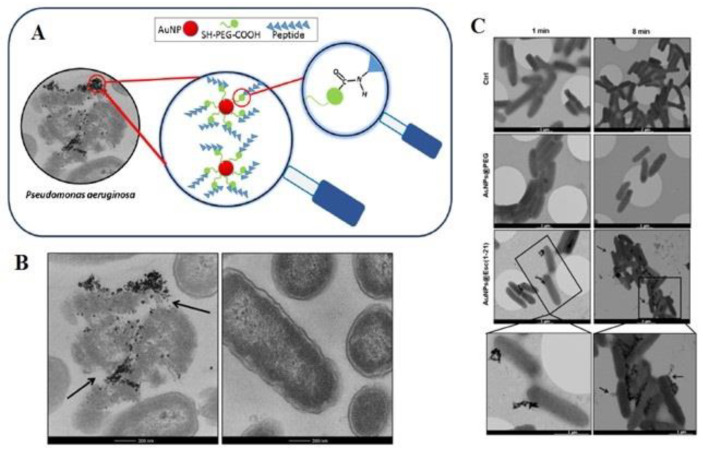
(**A**) Image of Esculentin-1a (1-21)-NH2 coated Au NPs and its interaction with bacteria. (**B**) Treatment of *P. aeruginosa* with (**left**) Au NPs@Esc(1-21) and (**right**) buffer as a control. (**C**) Schematic of *P. aeruginosa* cells treatment with Au NPs@Esc(1-21), Au NPs@PEG, and buffer [114]. Reprinted with permission from Elsevier.

The conjugation of AMP with Au NPs via a polyethylene glycol (PEG) linker shows the high anti-*P. aeruginosa* activity of free esculentin-1a through 15-fold without being toxic to human keratinocytes [114]. In this study, researchers used frog skin AMP esculentin-1a(1-21)NH2 with a durable activity against clinical samples of *P. aeruginosa*. If Eu (1-21) Au NP was used against *P. aeruginosa*, these nanoplatforms showed their antibiofilm properties, and almost half of the biofilm was destroyed within 2 h of treatment with the peptide concentration. The results indicate the high antibacterial activity of AMP esculentin-1a, which binds to Au NPs through PEG, then free peptides. This is likely according to the significant amount of Esc (1-21) present in the Au NPs at the PEG surface, nevertheless due to its orientation, and the great concentration of Au NPs@Esc (1-21) in the bacterial surface in addition to its long-term bioavailability. As a result, Au NPs@Esc (1-21) illustrate a disruption of the membrane as a significant assay for killing microbes [114] (Figure 3).

Au NPs can also be used in AMP delivery and validate the significance of their roles in gene and drug delivery applications. The conjugation of Au NPs with DNA aptamer shows effectively delivered AMPs into mammalian cells to prevent *S. typhimurium* colonization in mouse subjects [115]. Additionally, the conjugation of AMPs with Au NPs can bring together genes into stem cells [116].

**Figure 4:**
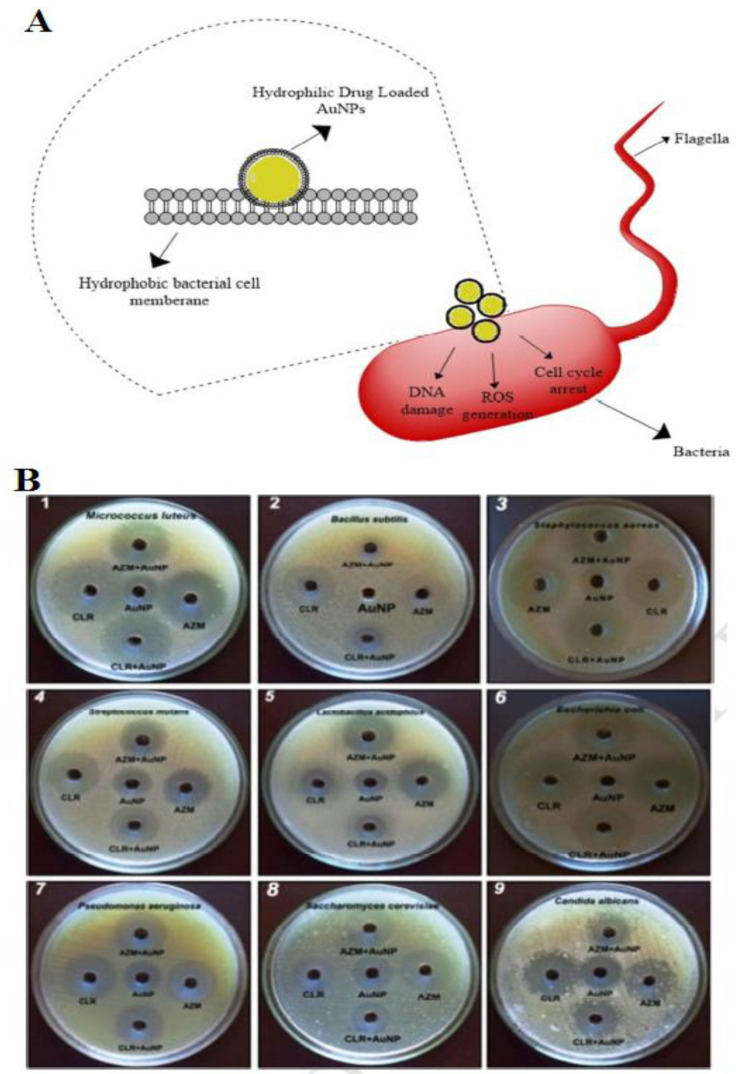
Schematic illustration of the killing of bacterial cells by Au NPs (**A**); schematic of spectrum antimicrobial activity of Au NPs, naked drug, and drug-loaded Au NPs against oral pathogenic pathogens. (**B**) The AuNPs (100 µg) and drug-AuNPs show a wide-spectrum antimicrobial effect against the pathogens. AuNPs exhibited antimicrobial activity through the creation of a zone of inhibition ranging from about 17 mm against pathogens excluding *B. subtilis*, which shows less of a response with a 9 mm zone [117]. Reprinted with permission from Elsevier.

In general, about 70 fungi and bacteria are described to be related to oral disease. Au NPs with a size of approximately 30 nm synthesized by green assay through *Justicia glauca*, displays an antagonistic outcome with Clarithromycin and Azithromycin antibiotics in contradiction of oral pathogenic bacteria and fungi, such as *Streptococcus mutans, Bacillus subtilis*, *Staphylococcus aureus*, *Saccharomyces cerevisiae*, *Lactobacillus acidophilus*, and *E. coli*. The Au NPs and drug conjugated Au NPs exhibited possible antimicrobial action against the oral pathogens. Minimum Inhibitory Concentration (MIC) principles of Au NPs were detected in the range of about 6–25 µg/mL against oral pathogens. Bacterial cell membranes have hydrophobic properties that prepared glycoproteins and phospholipids may help drug-loaded Au NPs transfer through the membrane. In fact, the interaction of NPs with bacteria can inhibit the growth of bacteria through the growth signaling pathway. These green synthesized Au NPs show the high efficiency of anti-microbial activity of NPs in AMR (Figure 4A) [111,117,118,119].

The use of nanomaterials such as Au NPs, silica NPs, etc. for controlled antibiotic delivery have several advantages including the reduction of toxicity, drug solubility improvement, and lower antibiotic dosage [120,121]. The preparation of carriers containing Au–silica core-shell mesoporous NPs (Au@MNs) and silica mesoporous NPs, loaded with amoxicillin (Amox) and ofloxacin was applied against *P. aeruginosa, E. coli,* and MRSA. The loading potential of these nanocarriers was assessed towards ofloxacin and amoxicillin antibiotics, and a great encapsulation of 70% (Au@MNs) and 62% (MNs) were reached in comparison to amoxicillin. Both Au@MNs and silica mesoporous NPs presented an important role in the actual delivery of amoxicillin for *P. aeruginosa* and MRSA. Decreases of 10 times (Amox@MNs) and 20 times (Amox@Au@MNs) in the quantity of antibiotics to *P. aeruginosa*; and a decrease of about 20 times (Amox@MNs) to MRSA was associated with a decline of resistance and finds these carriers capable of attacking AMR [122].

Since the unintended constant contact of bacteria with antibiotics in the environment increases AMR, one of the solutions in the fight against AMR is the detection of antibiotics in the environment that can be used to treat bacterial infections in humans. AMR in humans is powered through empirical infection diagnostics and unintended antibiotics revelation [123].

**Figure 5:**
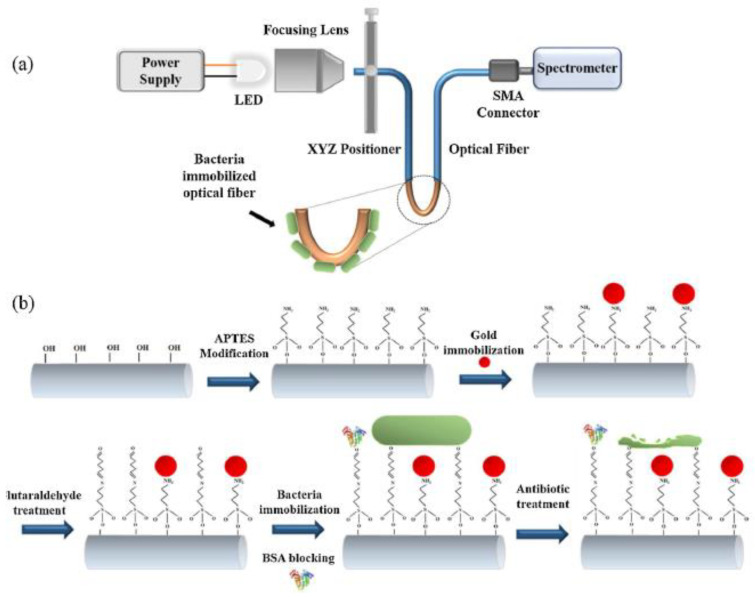
Schematic of an optical and sensing detection assay for the third generation of antibiotics through bacterial cells, (**a**) Optical detection assembly (**b**) The biosensing mechanism of antibiotics with bacterial cells [123]. Reprinted with permission from Elsevier.

Au NPs have been recognized as outstanding properties in LSPR biosensors according to the spectral response to the NPs surface, and the simplicity of observing the signal of light according to their absorption and durable scattering [124]. An optical fiber biosensor based on LSPR for the capture of *P. aeruginosa* and *E. coli* monitored by antibiotic refereed lysis has been revealed in rapid drug susceptibility testing. *P. aeruginosa* RS1 may be sensitive to the third generation of ceftazidime, cephalosporins, and cefotaxime. *P. aeruginosa* can be immobilized on Au NPs in the quantification of cephalosporins with a range of 0.01–1 μg/mL and LOD was established to be 0.5 μg/mL in human serum. This biosensor can act as a substitute to mass spectrometry and chromatography methods for the cheap and rapid detection of antibiotics using the optical properties of Au NPs (Figure 5) [123].

Au is an inert and rare metal to microbes. However, ionic Au (Au+ and Au3+) shows high antimicrobial activities. The antibacterial effect of ionic gold against various bacteria such as *Salmonella typhiurium*, *S. aureus,* and *E. coli* was shown by Dasari and colleagues [125]. Au^+^ with an IC_50_ range of about 0.30–0.50 µM and Au^3+^ with IC_50_ range of 0.320–0.50 µM were found to be greatly toxic to these bacteria. Ionic gold shows antimicrobial activity against *P. aeruginosa* with various AMR profiles. The combination of disc diffusion assays with the smallest inhibitory/bactericidal concentrations (MIC/MBC) were used to regulate the antimicrobial efficiency of ionic gold. Ionic gold at increasing concentrations shows improved antimicrobial activity and antimicrobial values against all strains tested. In fact, ionic gold can be used as a different antimicrobial agent against AMR *P. aeruginosa* [126]. Au NPs are modified, and these modified Au NPs can improve their antibacterial properties in antibacterial use. They can also be used as drug nanocarriers to enhance drug efficiency, and their thermal properties can be assembled to kill microbes.

The photothermal and photodynamic properties of AuNPs are a foundation in their medical applications [127]. Photothermal therapy (PTT) acts through irradiating Au NPs using a laser source to create heat. This heat damages neighboring bacterial cells [128]. Gold nanorods (AuNRs) and nanostars (AuNSTs) are examples applied in the PTT assay in biofilm abolition [129,130]. An antibacterial PTT using a phospholipid-decorated gold nanorod (DSPE-AuNR) was developed against *P. aeruginosa* biofilm. Results exhibited about a 6 log cycle reduction of the viable bacterial count with the treatment of *P. aeruginosa* with DSPE-AuNR after laser irradiation. The results show important changes in the shape of the bacterial membrane using the DSPE-AuNR. These results suggest that the heat produced with DSPE-AuNR excitation is accountable in the lysis of bacteria in biofilm cultures, though the probable contribution of the AuNRs themselves to the detected antibacterial effect should not be omitted [131].

In another study, the antibacterial activity of a chitosan-based hydrogel with embedded gold nanorods (Ch/AuNRs) under laser irradiation was developed. The antibacterial activity was evaluated on MDR strains. The results exhibited antibacterial activity of the Ch/AuNRs with MICs of 4 µg/mL, and low toxicity with cell viability of 80% after testing against a model of macrophage cells. This antibacterial activity is an effect of singlet oxygen production as well as the estrangement and the autolysis of bacterial membranes [132].

The macrophage membrane-coated system can be applied in medicine by bacterial pretreated macrophage membranes in precise bacterial recognition. A system was established through research by which *S. aureus* treated macrophage membranes were connected to Au–Ag nanocages making the conjugate Sa-M- GSNC. This nanosystem presented several benefits such as: 1. The capability to adhere to bacterial cells in therapy; 2. The use of the photothermal effect, which caused considerably decreased bacterial counts; and 3. High biocompatibility and blood circulation time when established on mice [133].

**Figure 6:**
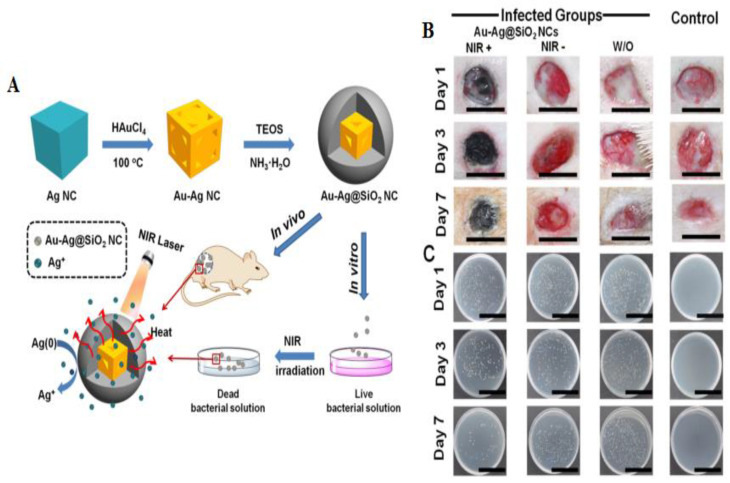
Schematic illustration of the Silica-coated Au-Ag nanocages displays antibacterial activity through photothermal activity (**A**). Schematic of wound tissues with various treatments on three days (**B**). Photographs of bacterial colonies from tissues of various treatment groups (**C**) Reprinted with permission from [134]. Copyright 2022 American Chemical Society.

Silica-coated Au-Ag nanocages under NIR laser exhibited improvements in bacterial resistance. Coating the Au–Ag nanocages with silicon dioxide enhanced the plasmon resonance of the Au–Ag nanocages (Figure 6). In this study, under irradiation of Au–Ag@SiO_2_ nanocages with a NIR laser, there was a temperature increase. It was observed that the rise in the concentration of nanocages and the time of irradiation was associated with an increase in temperature. The results indicated that the heat formed through this nanoplatform was quick and capable of removing *S. aureus*, *Enterobacter* spp, *K. pneumoniae,* and *P. aeruginosa* [134].

**Figure 7:**
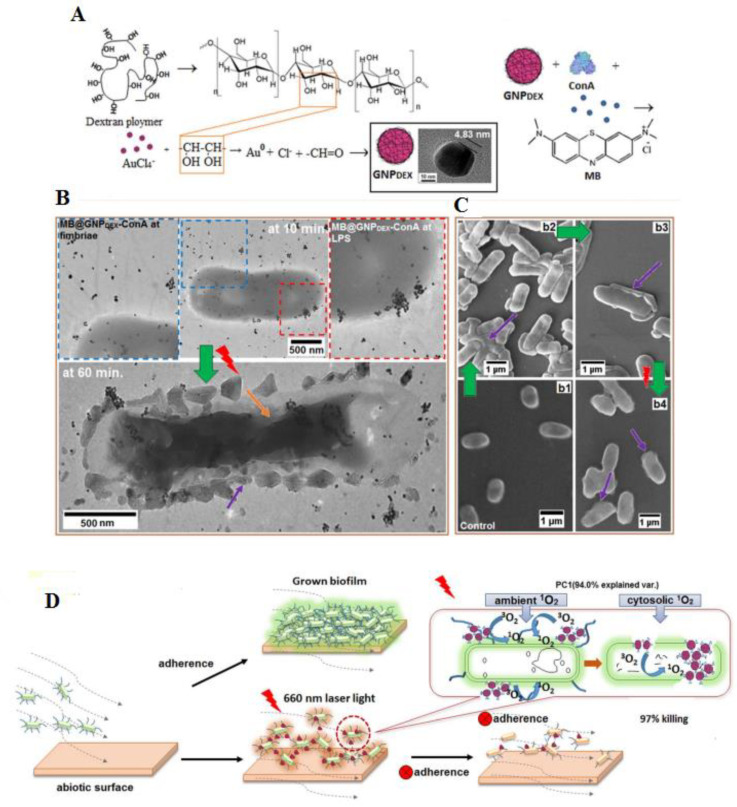
Schematic representation of the synthesis of dextran capped AuNPs (**A**). TEM image of localized MB@GNPDEX-ConA on *K.Pneumoniae*-12 bacterial surface and treatment. The schematic displays the cytological mass with accumulated conjugates (yellow arrow) and cell surface discomposure (**B**). The uniform morphology of bacterial aggregation and control cells according to concanavalin A (ConA)-mediated connection of conjugates (**C**). With light irradiation, the singlet oxygen produces ambient ^1^ and cyto O_2,_ however, the cytosolic singlet oxygen influence the bactericidal effect and the absence of adherence through ambient oxygen. Singlet oxygen around the bacterial cells decreases the adherence ability (**D**) [135] Reprinted with permission from Elsevier. Table 2 displays other examples of the application of Au NPs in antimicrobial resistance activity (AMR).

Researchers established a photodynamic antibacterial delivery system comprised of ConA directed dextran-capped Au NPs (GNPDEX-ConA) conjugated to PS MB establishing the MB@GNPDEX-ConA. This platform enhanced the efficiency of MB-induced killing of MDR clinical isolates, such as *Klebsiella pneumoniae* (*K. pneumoniae*), *E. coli*, and *Enterobacter cloacae*. With photothermal activation, this method was capable of destroying about 95% of MDR bacteria and exhibited no toxic effects as confirmed with HEK293 cells. ConA and dextran assisted in the connection of this conjugate to the bacterial surface and then bacterial surface lipopolysaccharides, as revealed in Figure 7. Single oxygen created through the conjugated MB after photoactivation was mostly accountable in the bacterial abolition. The results showed that the existence of MB in near contiguity to the Au NPs improved the production of singlet oxygen and improved the antibacterial abilities [135].

## 5. Challenges of Ag and Au NPs as Antibacterial Nanomedicines

The development of Ag and Au-based antimicrobial therapeutics is a complicated subject since the acknowledgment of various silver resistant strains in recent years [14,148]. Ag and Au NPs can be applied as antibacterial agents to afford or transport drugs in a directed manner and can solve critical complications, including multidrug resistance [14]. Multifunctional Ag and Au-based materials can be applied in combination therapy [149]. Metal-NPs can use their antibacterial activity through various mechanisms, including the interaction with the bacterial cell wall, inhibition of biofilm creation, production of ROS, and stimulation of intracellular effects [148].

Although Ag NPs have high antibacterial activity, the cytotoxicity of silver ions released through Ag NPs and their accumulation in the body may limit their applications in nanomedicine [150]. The Ag NPs also show toxicity against hepatocytes and fibroblasts or bone-marrow cells [151]. Bondarenko et al. showed that MIC values in bacteria in Ag and ZnO NPs are 7 and 500 mg/L, respectively [152]. Researchers developed porous Ag-Au nanosheets with lower cytotoxicity that had antibacterial properties similar to *S. aureus* strains [153]. The surface and size of NPs affect their toxicity [154]. Because of their small size, NPs can be transported to the systemic circulation after inhalation, skin contact, or oral administration. When NPs are in the bloodstream, they come in contact with various components of the blood and may disrupt the normal function of platelets, causing bleeding or thrombosis. The biocompatibility of NPs with blood elements is a controversial issue [155]. Overcoming MDRs has evolved to improve antibiotics, and as a result, nanomedicine delivery systems can provide ways to develop antimicrobial nanomedicine. The use of biological methods, such as greener assays or the use of greener solvents, are also probable to be useful both in the field of environmental remediation and in other significant areas [156].

The biocompatibility and easy synthesis of Au NPs have made them suitable candidates for medical applications, but whether they can be affected by intracellular agents remains to be seen. In fact, the small size of Au NPs enhances their antibacterial effects; these NPs are easily modified and they have significant antibacterial applicability [27]. As a result, Au NPs can be modified to increase their antibacterial properties in antibacterial clinical use, however, more research is needed to investigate the toxicity in the living environment after long periods and high doses. Despite various benefits, Au NPs have significantly lower antibacterial activity than Ag NPs [157]. Long-term in vivo safety and experimental explanations are important in transporting advanced therapeutics into the clinical field and in regulating their development. The safety of Au NPs was also evaluated through the healthy performance of mice and the lack of toxicities in the spleen and liver. Researchers performed a comparison to investigate the biological distribution of PEGylated-AuNPs vs. AuNPs alone with diameters ranging from 20 nm to 50 nm. They showed that at 2 days after injection, accumulation in the liver and spleen was significantly reduced by PEGylation [158]. Another field where we consider needed development is in the standardization of experiments to gain comparable studies. We hope in the future to have effective and safe nanogold-based therapeutics where gold’s abilities are proven to eliminate the threatening superbugs in the world. The comparison between the advantages and limitations of both Ag and Au NPS and the challenges ahead are given in Table 3.

According to the literature, another of the challenges that can be mentioned in the use of metals, especially heavy metals, such as copper, arsenic, cadmium, and thallium, is their effect in inducing an increase of antibiotic-resistant bacteria (ARBs) in water sources or the environment, and changes in antibiotic resistance. In addition to heavy metals, the combination of heavy metals and other noble metals, such as Au and Ag, can cause combined pollution happenstances. Pre-treatment of surface water can be a significant source of ARBs and antibiotic resistance genes (ARGs) and therefore determinants of antibiotic resistance in drinking water systems. Studies have shown that the resistance of bacteria to heavy metals and antibiotics can increase simultaneously with the existence of heavy metals. As a result, the presence of heavy metals can change the antibiotic resistance of water sources and indirectly increase the threat to human health [159,160]. More considerations are proposed to be remunerated on the environmental and safety complications. However, biosorbents can be used in heavy metal removal. These biosorbents exhibit elegant prospects as low-cost investments to protect the environment from pollution. To advance a constant biosorption procedure, more studies are required in biosorbent properties, in terms of zeta potential, the morphology of surface, and particle size, as these are significant in biosorption tests [161].

ARGs have the possibility to be transmitted through varied environments. Increased antibiotics have triggered horizontal gene transfers (HGT) in widespread ARGs in the environment. In fact, HGT permits bacteria to exchange their antibiotic resistance genes between varied species. The application of ultraviolet (UV) technology, in combination with other oxidants, peroxymonosulfate, and photocatalysts to engage the high reactivity of created reactive species, can be used to enhance the degradation efficacy of ARGs and decrease the migration of ARGs during the inactivation of microbes [162,163].

## 6. Conclusions and Future Direction

AMR is a major threat in social and hospital settings and leads to many deaths. Trying to discover novel drugs to stop the development of drug resistance, therefore, is not a technological approach to restraint and control. AMR is corroding our capacity to control infections with current antibiotics and there is a need to develop new methods to combat it. Innovation and the development of metal NPs such as Au and Ag, due to their special antimicrobial properties, can be used as new tools to address this public health threat. Metal nanoparticles, such as Ag, can kill bacteria and prevent the formation of biofilms.

Nanomedicine research provides easy access to innovative and nano symmetric methods that ultimately enable us to design and build intelligent drug delivery systems with low toxicity and high efficiency that can be realized from the unique benefits of nanomedicines; from toxicity reduction to high efficiency, successful trials, and significant market outlook. In addition, the development of controlled drug/antibiotic delivery systems based on nanostructures such as Au NPs can be a very attractive and effective way to overcome AMR. However, one of the most important issues is the discussion of toxicity effects that make their use in clinical applications difficult. In addition, the antimicrobial effect of NPs is dose-dependent and requires the use of high concentrations of NPs above the MIC to achieve robust treatment results, along with potential toxicity. As a result, there are limitations on the dose of nanomaterial agents that limit their work in vivo and clinical systems. In addition to the bacterial cell membrane, the amount of ROS produced by bacterial cells can also damage the host cell’s membranes. In general, more studies are needed using in vitro and in vivo models to ensure that more NPs can be used. Another way to combat AMR is to identify the risks associated with antibiotic-resistant bacteria in the environment or contaminated soils. This means that integrated and accurate assays are needed to be able to simultaneously identify different analytes, including antibiotic and pathogenic microbes. Utilizing the optical properties of metal NPs, including Au NPs, in combination with other polymers and other nanomaterials can be a promising choice for the simultaneous detection of complex biological and chemical mixtures in soil, as well as the development of optical and electrochemical biosensors. Therefore, using biosensors can provide accurate and rapid diagnoses and minimize the risk of AMR emissions into the environment.

## Figures and Tables

**Table 1:** Other examples of the application of Ag NPs in antimicrobial resistance activity (AMR).

Bacteria	Type of NPs	Size (nm)	Description	Ref.
*E. coli*	Ag NPs	30 nm	The EC50 of the Ag NPs against *E. coli* was about 11 mg/L.	[94]
*E. coli* and *Vibrio cholerae*	Ag NPs	~30 nm	Ag NPs were verified as the MIC against *V. cholerae* and *E. coli.*	[95]
*P. aeruginosa, E. coli*, *S.aureus*	Ag NPs	140 nm	Ag NPs show antimicrobial activity against gram-positive (1500 ppm) and gram-negative bacteria (125 ppm.)	[96]
*K. pneumoniae*	Ag NPs	5.2 ± 1.2 nm	Ag NPs can induce triclosan-like antibacterial action against *K. pneumonia.*	[97]
*S. marcescens*, *P. aeruginosa*, *C. violaceum*	Ag NPs	5–20 nm	The Ag NP inhibition rate of 87%, 81%, and 71% for biofilms of *C. violaceum*, *S. marcescens*, and *P. aeruginosa*, respectively.	[98]
*E. coli* and *Staphylococcus aureus*	Ag NPs	12 nm	Ag NPs show a great antimicrobial activity against *E. coli*, with a MIC of about 120 µmol/L.	[99]
*S. aureus*	Ag NPs	27 nm	The green synthesis of Ag NPs can be used for the inhibition of *S. aureus.*	[100]
*P. aeruginosa, E. coli*	TGA-stabilized AgNPs	16–25 nm	Vancomycin-AgNPs can improve antibacterial activity against gram-positive bacteria.	[101]
*S. pneumoniae*	Ag NPs	63.65 ± 12.71 nm	The NP coated antimicrobial medical devices to battle against MDR infection.	[102]
*P. aeruginosa*	Ag NPs	~2.4, 13.92 nm	The NPs can be used to evade multidrug efflux pumps.	[103]
*A. baumannii*	TMCN-Ag NPs	Less than 60 nm	The NPD connect to the cell wall causing changes in the permeability of the cell membrane.	[104]

MIC: Minimum inhibitory concentration, NPs: Nanoparticles, TMCN: Trimethylchitosan.

**Table 2:** Other examples of the application of Au NPs in antimicrobial resistance activity (AMR).

Bacteria	Type of NPs	Size (nm)	Description	Ref.
*S. aureus*, *E. coli*	Au NPs	6–60 nm	Au NPs show good antimicrobial activity against *S. aureus* compared to *E. coli.*	[136]
*P. aeruginosa* PPAO1, *Serratia marcescens*	Au NPs	19.97 nm	Au NPs can inhibit the biofilm formation and EPS creation of both bacteria.	[137]
*St. aureus*	Au NPs	12 nm (aptamer-Au NPs)	Detection of *S*. *aureus* by Van-modified Au NCs and aptamer-modified Au NPs in 30 min with LOD was 10 CFU mL^−1^.	[138]
*P.aeruginosa*, *E. coli*	Au NPs	NA	Inhibition of efflux pump by Emb-chi- Au NPs.	[139]
*P. acnes, S. aureus*	Au nanorods	Length and width of ~49 nm and ~12 nm	Au nanorods showed a greater MIC than unpurified ones, which shows that impurities have a chief effect on the antibacterial activity.	[140]
*E. coli, S. aureus*	Au nanostars modified with various thiol groups	Central core diameter of about 18 nm, branches with a length of 12 nm	About 99% of the bacterial strains were removed after the photothermal effect.	[141]
*S. aureus, E. coli, P. aeruginosa*	Thiol chitosan-wrapped Au nanoshells (TC-AuNSs)	~185 nm	TC-AuNSs were capable of destroying bacteria inside a short time of NIR laser irradiation.	[142]
*E. coli*, *S. aureus*	Au NPs	~2 nm	More than a 3-log reduction in viable *S. aureus* after 6 h of light exposure.	[143]
*E. coli*, *K. pneumoniae* and *Enterobacter cloacae*	Dextran-capped Au NPs	~23 nm	MB@GNPDEX-ConA mediated treatment against various multi-drug resistant infections with 97% killing of bacteria.	[135]
*S. epidermidis*, *S. aureus*, *B. subtilis MDR S.epidermidis*	Aminosacharrides D-glucosamine (GluN), D-mannosamine (ManN), D-galactosamine (GalN) Au NPs	~4 nm	AuGluN exhibited the greatest antibacterial activity with MIC of less than 4 µg/mL.	[144]
*St.aureus*, *E. coli*	Au NPs	2–5 nm	PU- Au NPs-CV shows antibacterial surfaces were attained by 1 mg/mL swell encapsulation concentrations of 2 nm Au NPs.	[145]
*E. coli*	Colistin –Au NPs	5 nm	Delivering Colistin through Au NPs exhibited a reduction in the MIC against *E. coli* with about a 6-time reduction detected compared to antibiotics alone.	[146]
*S. aureus*	Gold nanostars (AuNSTs), gold nanoflowers (AuNFs)	AuNSTs: ~26 nm, AuNFs: ~40 nm	AuNSTs and AuNFs triggered a reduction in the growth rate of bacteria by ~60% and 76%.	[147]

MIC: Minimum inhibitory concentration, LOD: Limit of detection, NCs: Nanoclusters, NPs: Nanoparticles, Emb: Embelin, Chi; Chitosan, CV: crystal violet, and PU: polyurethane.

**Table 3:** Advantages and limitations of antibacterial Ag and Au NPs.

Advantages	Limitations
Crosses tissue barriers	Toxicity
Controlled drug release	Accumulation if intravenously injected
Production of ROS	High systemic acquaintance to administered drugs
Antibacterial effect	Need equipment for mass production
Photothermal effect	
Low immunosuppression	
Easy synthesis	

## Data Availability

The data presented in this study are available on request from the corresponding author.
