# Correction: Rabiee et al. Silver and Gold Nanoparticles for Antimicrobial Purposes against Multi-Drug Resistance Bacteria. *Materials* 2022, *15*, 1799

**DOI:** 10.3390/ma17122841

**Published:** 2024-06-11

**Authors:** Navid Rabiee, Sepideh Ahmadi, Omid Akhavan, Rafael Luque

**Affiliations:** 1 Department of Physics, Sharif University of Technology, Tehran 11155-9161, Iran; oakhavan@sharif.edu; 2 School of Engineering, Macquarie University, Sydney, NSW 2109, Australia; 3 Student Research Committee, Department of Medical Biotechnology, School of Advanced Technologies in Medicine, Shahid Beheshti University of Medical Sciences, Tehran 19857-17443, Iran; speahmadi@yahoo.com; 4 Cellular and Molecular Biology Research Center, Shahid Beheshti University of Medical Sciences, Tehran 19857-17443, Iran; 5 Departamento de Química Orgánica, Campus de Rabanales, Universidad de Córdoba, Edificio Marie Curie (C-3), Ctra Nnal IV-A, Km 396, E14014 Cordoba, Spain

Following publication [1], the authors raised concerns to the editorial office relating to certain terminology and the relevance of several citations contained within this publication. The editorial office then completed an investigation adhering to our Updating Published Papers policy (https://www.mdpi.com/ethics#_bookmark30). As a result of the investigation and discussions between the authors and the editorial office, it was decided to issue a correction containing the following changes:

**Remove/Replace Citations**

The original publication contained the following irrelevant citations that have now been removed and the references have been renumbered: [2], [4], [8], [9], [10], [12], [13], [15], [17], [18], [19], [26], [27], [28], [30], [37], [38], [39], [42], [49], [50], [53], and [65].

In addition, the following references were replaced: [22], [23], [31], [32], [33], [34], and [35].

The list of updated and rearranged references is as follows:

[22]: Alavi, M.; Rai, M. Recent advances in antibacterial applications of metal nanoparticles (MNPs) and metal nanocomposites (MNCs) against multidrug-resistant (MDR) bacteria. *Expert Rev. Anti-Infect. Ther.* **2019**, *17*, 419–428.

[23]: Mishra, A.; Pradhan, D.; Halder, J.; Biswasroy, P.; Rai, V.K.; Dubey, D.; Kar, B.; Ghosh, G.; Rath, G. Metal nanoparticles against multi-drug-resistance bacteria. *J. Inorg. Biochem.* **2022**, *237*, 111938.

[31]: Ebrahim-Saraie, H.S.; Heidari, H.; Rezaei, V.; Mortazavi, S.M.J.; Motamedifar, M. Promising antibacterial effect of copper oxide nanoparticles against several multidrug resistant uropathogens. *Pharm. Sci.* **2018**, *24*, 213–218.

[32]: Ye, Q.; Chen, W.; Huang, H.; Tang, Y.; Wang, W.; Meng, F.; Wang, H.; Zheng, Y. Iron and zinc ions, potent weapons against multidrug-resistant bacteria. *Appl. Microbiol. Biotechnol.* **2020**, *104*, 5213–5227.

[33]: El-Kattan, N.; Emam, A.N.; Mansour, A.S.; Ibrahim, M.A.; Abd El-Razik, A.B.; Allam, K.A.; Riad, N.Y.; Ibrahim, S.A. Curcumin assisted green synthesis of silver and zinc oxide nanostructures and their antibacterial activity against some clinical pathogenic multi-drug resistant bacteria. *RSC Adv.* **2022**, *12*, 18022–18038.

[34]: Basavegowda, N.; Baek, K.H. Multimetallic nanoparticles as alternative antimicrobial agents: challenges and perspectives. *Molecules* **2021**, *26*, 912.

[35]: Ranpariya, B.; Salunke, G.; Karmakar, S.; Babiya, K.; Sutar, S.; Kadoo, N.; Kumbhakar, P.; Ghosh, S. Antimicrobial synergy of silver-platinum nanohybrids with antibiotics. *Front. Microbiol.* **2021**, *11*, 610968.

**Text Correction**

In addition, two phrases in the original publication were not appropriate. The authors would like to change “vegetative electron microscopy” to “scanning electron microscopy” and “extracellular cells” to “extracellular membrane”. A correction has been made to Section 3. Ag Nanoparticles for Antimicrobial Resistance, paragraph 1.

The authors state that the scientific conclusions are unaffected. This correction was approved by the Academic Editor. 
